# Assessment of portal image resolution improvement using an external aluminum target and polystyrene electron filter

**DOI:** 10.1186/s13014-019-1274-4

**Published:** 2019-04-25

**Authors:** Jonggeun Baek, Hyungdong Kim, Byungyong Kim, Youngkee Oh, Hyunsoo Jang

**Affiliations:** 1 Department of Radiation Oncology, Dongguk University Gyeongju Hospital, Gyeongju, South Korea; 2 Department of Radiation Oncology, Daegu Fatima Hospital, Daegu, South Korea; 3 Department of Radiation Oncology, Semyung Christianity Hospital, Pohang, South Korea; 4 Department of Radiation Oncology, Keimyung University College of Medicine, Daegu, South Korea; 5 Department of Radiation Oncology, Dongguk University School of Medicine, Gyeongju, South Korea; 6 Department of Medical Sciences, Radiation Oncology, Graduate School of Ajou University, Suwon, South Korea

**Keywords:** Low-Z target, Portal imaging, Spatial resolution, Contrast resolution, Contrast and details

## Abstract

**Background:**

In this study, an external 8 mm thick aluminum target was installed on the upper accessory tray mount of a medical linear accelerator head. The purpose of this study was to determine the effects of the external aluminum target beam (Al-target beam) on the portal image quality by analyzing the spatial and contrast resolutions. In addition, the image resolutions with the Al-target beams were compared with those of conventional 6 megavoltage (MV) images.

**Methods:**

The optimized Al-target beam was calculated using Monte Carlo simulations. To validate the simulations, the percentage depth dose and lateral profiles were measured and compared with the modeled dose distributions. A PTW resolution phantom was used for imaging to assess the image resolution. The spatial resolution was quantified by determining the modulation transfer function. The contrast resolution was determined by a fine contrast difference between the 27 measurement areas. The spatial and contrast resolutions were compared with the those of conventional portal images.

**Results:**

The measured and calculated percentage depth dose of the Al-target beam were consistent within 1.6%. The correspondence of measured and modelled profiles was evaluated by gamma analysis (3%, 3 mm) and all gamma values inside the field were less than one. The critical spatial frequencies (*f*_50_) of the images obtained with the Al-target beam and conventional imaging beam were 0.745 lp/mm and 0.451 lp/mm, respectively. The limiting spatial frequencies (*f*_10_) for the Al-target beam image and the conventional portal image were 2.39 lp/mm and 1.82 lp/mm, respectively. The Al-target beam resolved the smaller and lower contrast objects better than that of the MV photon beam.

**Conclusion:**

The Al-target beams generated by the simple target installation method provided better spatial and contrast resolutions than those of the conventional 6 MV imaging beam.

## Introduction

A portal image is defined as a patient image taken with a therapeutic megavoltage (MV) beam to verify patient positioning and target localization [1]. Portal images obtained with MV portal imagers have been used in patient positioning and target localization over the past 20 years and require short image acquisition times. However, MV portal images have a poor quality because the dominant primary attenuation mechanism in the MV photon energy range is the Compton interaction [1–3]. With the advent of kilovoltage (kV) on-board imagers (OBI), many institutions are performing patient setup verification with high-resolution soft tissue information [4–6]. However, kV-OBI systems have several drawbacks, such as geometric misalignment of the isocenters between treatment and imaging, image artifacts produced by high-Z materials, greater maintenance costs, increased quality assurance, and the absence of beam’s eye viewing [2, 7–9]. Thus, kV-OBI systems cannot completely replace MV portal imaging systems, and MV portal imaging is invariably used when the OBI systems fail [10]. The kV-OBI systems and MV portal imaging systems should be complementary; however, the portal image quality should be improved.

In order to improve the portal image quality, the imaging beams should have a large fraction of diagnostic quality photons in the energy range of 25–150 keV [3, 11]. These useful low-energy photons are produced when the incident electron beam interacts with the target; however, most are removed within thick, high-Z target materials owing to photoelectric absorption. Moreover, low-energy photons transmitted through the target are absorbed by the flattening filter under the target, and therefore, only 0.3% of the photons remain in the portal imaging beam [2, 12]. Therefore, to generate a portal imaging beam that contains a large fraction of diagnostic quality photons, a thick and high-Z target and flattening filter should be eliminated from the linear accelerator (linac) head. In addition, a thin and low-Z target should be applied in the incident electron beam path, and the energies of the incident electron beams should be as low as possible in the current generation of medical linacs [13].

Multiple studies have shown that the thin, low-Z targets and lower incident electron beam energies are capable of producing large numbers of useful low-energy photons in the output spectrum of the portal imaging beam [1–3, 7, 13–15]. In the majority of the previous studies, low-energy photon beams have been generated by installing a low-Z target in the carousel of the medical linac head and modulating the electron beam energy. However, these mechanical modifications to the linac are complex and not applicable to existing linacs because without the assistance of the equipment manufacturer, modifications to the linac are not possible.

In this study, we aimed to improve the portal image quality by inserting an external low-Z target into the upper accessory tray mount without modifying the linac head configuration or modulating the electron energy. In a preliminary study, the external low-Z target material and thickness were determined by the Monte Carlo simulation, and an 8 mm thick aluminum target generated the greatest proportion of low-energy (25–150 keV) photons for a 6 MeV electron beam [10, 12].

The purpose of this study was to determine the effects of Al-target beams generated by the simple target installation method on the portal image quality by analyzing the spatial and contrast resolutions. The second aim of this study was to compare the image quality acquired with the Al-target beams to that of conventional 6 MV photon beam.

## Methods and materials

### A. Removal of the remaining incident electron beam

In a preliminary study, an 8 mm external aluminum target produced the largest proportion of useful low-energy photons for the 6 MeV electron beam than thick-optimized low-Z targets, such as 18 mm beryllium, 14 mm carbon, and 4 mm titanium [10]. The Al-target beam produced a total photon yield of 64.2% and a low-energy photon percentage among the total photons of 34.4%. This result was similar to that observed in a previous study, in which a low-Z target material was inserted in the linac head carousel, regarding the percentage of photons in the 25–150 keV range [2].

**Fig. 1:**
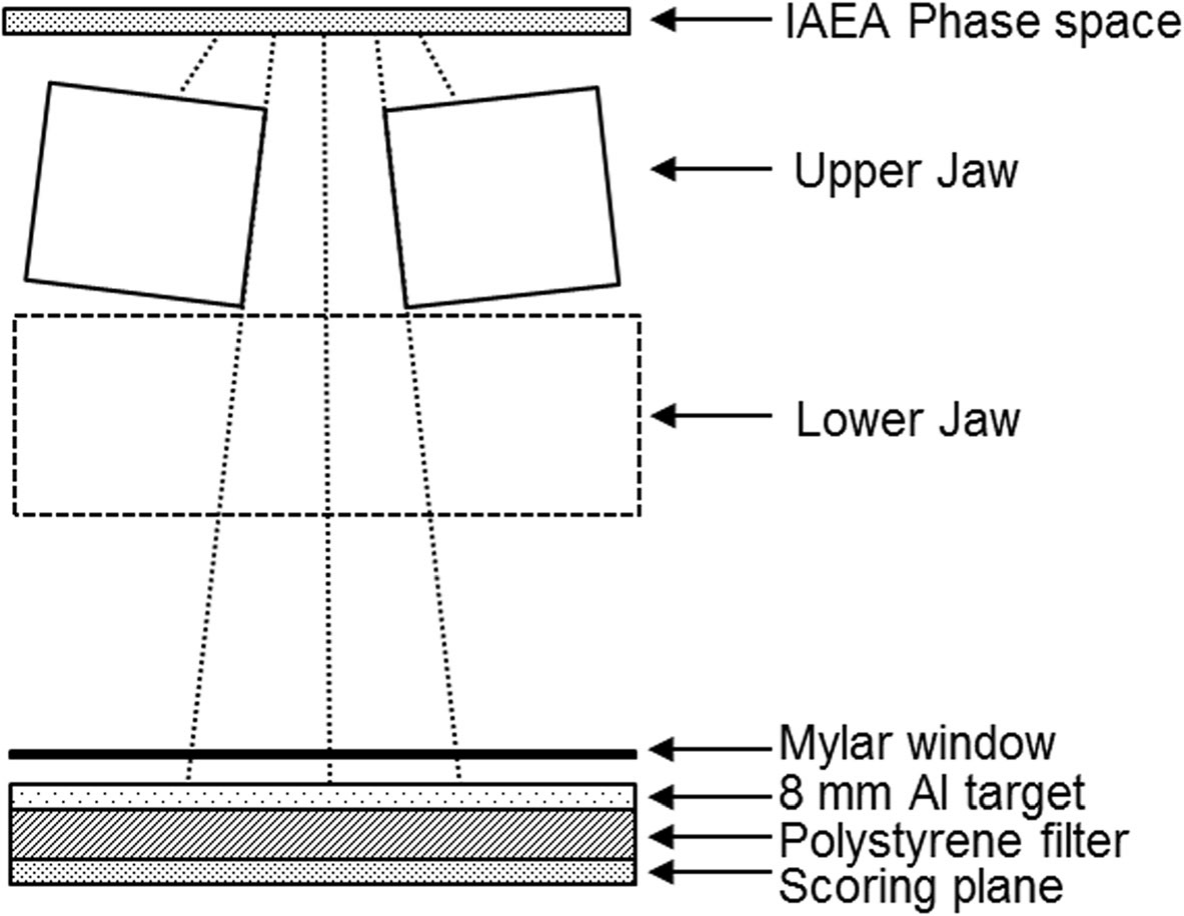
Schematic diagram of the medical linear accelerator components to generate the Al-target beams modelled using GEANT4 Monte Carlo simulation

However, 35% of the incident primary electrons remained in the Al-target beam. The electrons transmitted through the target caused noise in the portal images and increased patient exposure. Therefore, these remaining electrons should be removed. A polystyrene plate has been used as a filter to remove the electrons in previous studies, because polystyrene has a higher stopping power and lower photon beam generation efficiency than those of the higher Z materials for the electron energies of interest [2, 7, 16]. In this study, the GEANT4 Monte Carlo simulation was used to determine the optimal polystyrene filter thickness to maximize electron removal. As shown in Fig. 1, the polystyrene filter was located immediately below the aluminum target. The percentages of the remaining electrons, photons, and low-energy photons were analyzed by changing the polystyrene filter thickness from 4 to 20 mm. A phase space file provided by Varian was used as the beam source for the 6 MeV electrons, and an International Atomic Energy Agency (IAEA) format phase space file was generated using the GEANT4 code with the geometric input parameters of a medical linac [17–19]. The IAEA format phase space was recorded above the upper jaws, and the phase space was used as the beam source. The polystyrene filter and other linac head components, such as the upper and lower jaws, Mylar window, and 8 mm aluminum target, were modeled and simulated in the GEANT4 code. The field size was set at 20 × 20 cm^2^ at the isocenter. As shown in the Fig.1, the scoring plane was located immediately below the polystyrene filter, and the energy spectra of the particles were recorded and analyzed. The number of histories in the space file of the 6 MeV incident electrons was 7 × 10^9^, and the statistical uncertainty of the doses calculated by the Monte Carlo simulation was within 2%. The cut-off range of the photons, electrons, and positrons was 0.01 mm.

### B. Validation of the modeled aluminum target beam

To validate the accuracy of the Al-target beam modeled by the Monte Carlo simulation, the percentage depth dose (PDD) and lateral profiles were compared with the actual data obtained using the customized aluminum target and polystyrene filter. The measurements were performed using the Blue Phantom (IBA Dosimetry, Germany) and Farmer-type ion chamber with a volume of 0.64 cm^3^ (EXRADIN A12, Standing Imaging, USA). The PDD for the aluminum target beam was acquired for a field size of 20 × 20 cm^2^ at a source surface distance (SSD) of 100 cm in water. The lateral profile was measured at a maximum dose depth and field size of 20 × 20 cm^2^. The field sizes were set using linac jaws. To obtain the dose distribution by Monte Carlo simulation, a virtual water phantom was created using the same settings as in the actual measurement. The overall size of the water phantom was 50 × 50 × 50 cm^3^, and the voxel size was 5 × 5 × 5 mm^3^. The cut-off range of the photons, electrons, and positrons was 0.1 mm. The gamma factor analysis was used to evaluate the difference between the modelled and measured profiles. The measurement data set was used as the reference dose distribution, and the calculated data set is used as the evaluated dose distribution. The linear interpolation of evaluated dose distribution was conducted as 1 mm pixel (data point) spacing. The acceptance criteria of the gamma factor were based on the dose-difference of 3% and distance-to-agreement of 3 mm, respectively.

### C. Portal image acquisition

#### C.1 phantom

A PTW QC phantom (PTW, Freiburg, Germany) was used to assess the spatial and contrast resolutions owing to its simplicity and suitability for portal imaging applications. The spatial resolution was analyzed using 14 sets of brass lamella blocks and 11 spatial frequencies from 0.125 to 3.3 lp/mm, where lp represents a line pair, as listed in Table 1. The contrast resolution was analyzed using an aluminum test element with 27 holes of six different diameters and five different depths, as listed in Table 2.

**Table 1:** Specifications of the high-contrast elements in the PTW QC phantom

Dimensions (mm)	4	3	2	1.5	1	0.75	0.5	0.35	0.25	0.2	0.15
Resolution (lp/mm)	0.125	0.167	0.25	0.33	0.5	0.67	1	1.43	2	2.5	3.33

**Table 2:** Specifications of the low-contrast elements in the PTW QC phantom

Hole diameters (mm)	Hole depths (mm)				
15.0	0.5	1.0	2.0		
10.0	0.5	1.0	2.0	3.2	
7.0	0.5	1.0	2.0	3.2	4.8
4.0	0.5	1.0	2.0	3.2	4.8
2.0	0.5	1.0	2.0	3.2	4.8
1.1	0.5	1.0	2.0	3.2	4.8

#### C.2 detector

The computed radiography (CR, Agfa CR-85X system, Agfa Medical Systems, Ridgefield Park, NJ) system was used for portal image acquisition of the Al-target beam and 6 MV photon beam. The Al-target beam containing low-energy photons is not suitable for the electronic portal imaging device (EPID, Varian Medical Systems, Palo Alto, CA) owing to the permitted dose range (4–25 MV) of the detector and the copper build-up plate inside the device, which absorbs low-energy photons. The CR system can be implemented as digital images in both the Al-target beam and the 6 MV photon beam. Since the CR system used in our study has 2828 × 2320 pixels, the resolution of the acquired image using this system is higher than that of EPID with 1024 × 768 pixels. The CR system uses an imaging plate (IP) composed of a mixture of phosphors instead of the conventional film, which is enclosed in a light-tight CR cassette. The Al-target beams and 6 MV photon beams were irradiated to the CR cassette and the digital images were acquired through the CR reader.

#### C.3 experiment

The phantom was placed on the treatment couch, and the recessed lines on the sides of the phantom (in the horizontal and vertical directions) were aligned using the treatment room laser. The crosshairs in the field light were aligned with the lines on the surface plate of the phantom, and the field size was 26 × 26 cm^2^ at a 95.2-cm SSD. The CR cassettes were placed in the cassette holder under the couch, and the SID was set at 110 cm. The Al-target beam was generated in the 6 MeV electron mode with the customized aluminum target and polystyrene filter inserted into the upper accessory tray mount. All irradiations in both Al-target beam and 6 MV photon beam were performed using a Varian Clinac iX. The portal images for the 6 MV photon beams were acquired using 2 monitor unit (MU, 2.12 cGy) at a dose rate of 100 MU/min. The portal images for the Al-target beams were acquired using a 50 MU (0.20 cGy) at a high dose rate (1000 MU/min). Relatively large MUs and a high dose rate were applied to the imaging of the Al-target beam because of the low photon production efficiency (ratio of produced photons to incident electrons) of the Al-target beam generation process. The imaging doses for the two imaging beams in this experiment were comparable to those used by Roberts et al. [13].

### D. Image resolution analysis

All images acquired with the two imaging beams were imported to epidSoft software provided with the phantom for the quantitative analysis of the image contrast resolutions. All phantom images presented in this study were inverted to assess the image sharpness. No gray scale manipulation was performed.

#### D.1 spatial resolution

The spatial resolution demonstrates the level of detail that can be seen on an image and was quantified by determining the modulation transfer function (MTF). In the phantom, bar-pattern-type lamella blocks presenting spatial frequencies were used for determining the MTF. The MTF is calculated as the ratios of contrast k, where k is the ratio of object g(x) to image b(x’), as expressed by eqs. 1, 2.

1 $$ k=\frac{\max \left(g(x)\right)-\min \left(g(x)\right)}{\max \left(g(x)\right)+\min \left(g(x)\right)} $$

2 $$ k\mathrm{MTF}=\frac{\max \left(b\left({x}^{\prime}\right)\right)-\min \left(b\left({x}^{\prime}\right)\right)}{\max \left(b\left({x}^{\prime}\right)\right)+\min \left(b\left({x}^{\prime}\right)\right)} $$

The MTF can be expressed as follows.

3 $$ \mathrm{MTF}=\frac{\max \left(b\left({x}^{\prime}\right)\right)-\min \left(b\left({x}^{\prime}\right)\right)}{\max \left(b\left({x}^{\prime}\right)\right)+\min \left(b\left({x}^{\prime}\right)\right)}/\frac{\max \left(g(x)\right)-\min \left(g(x)\right)}{\max \left(g(x)\right)+\min \left(g(x)\right)} $$

Thus, MTF is calculated using the image contrast ratio as obtained using the maximum and minimum grayscale values, as shown in eq. (3). The calculated MTF results were normalized with respect to the smallest spatial frequency to obtain the relative MTF values. Further details of the derived formulas are provided by Coltman [20]. The spatial resolution properties of the two imaging beam modes were characterized by comparing the relative MTF curves and by quantifying the critical (*f*_50_) and limiting (*f*_10_) spatial frequencies. The critical spatial frequency was defined as the spatial resolution corresponding to 50% of the maximum relative MTF and was used to determine the image sharpness criteria. The limiting spatial frequency was defined as the spatial resolution corresponding to 10% of the maximum relative MTF and was used to determine the limiting resolution criteria [21].

#### D.2 contrast resolution (contrast-detail distribution)

The contrast resolution was determined by a fine contrast difference between the 27 measurement areas of different depths, diameters, and background areas around the measurement areas. The contrast differences were calculated using the following equation.

8 $$ \mathrm{contrast}\ \mathrm{difference}=\frac{\left|E-{E}_0\right|}{E_0}=1-{e}^{-\varDelta \mu t} $$

The parameters E and E_0_ are the relative energy amounts absorbed in the detector for the measurement and background areas. The parameter Δμ represents the attenuation coefficient difference between the two regions, and t represents the thickness of the measurement region. Therefore, the light output from the image is proportional to the energy absorbed. The contrast-detail distributions were quantified according to the degree of visualization of each hole in the low-contrast test element of the phantom, and the distributions were used to characterize the contrast resolution properties of the two imaging beam modes.

## Results and discussion

### A. Removal of the remaining incident electron beam

**Fig. 2:**
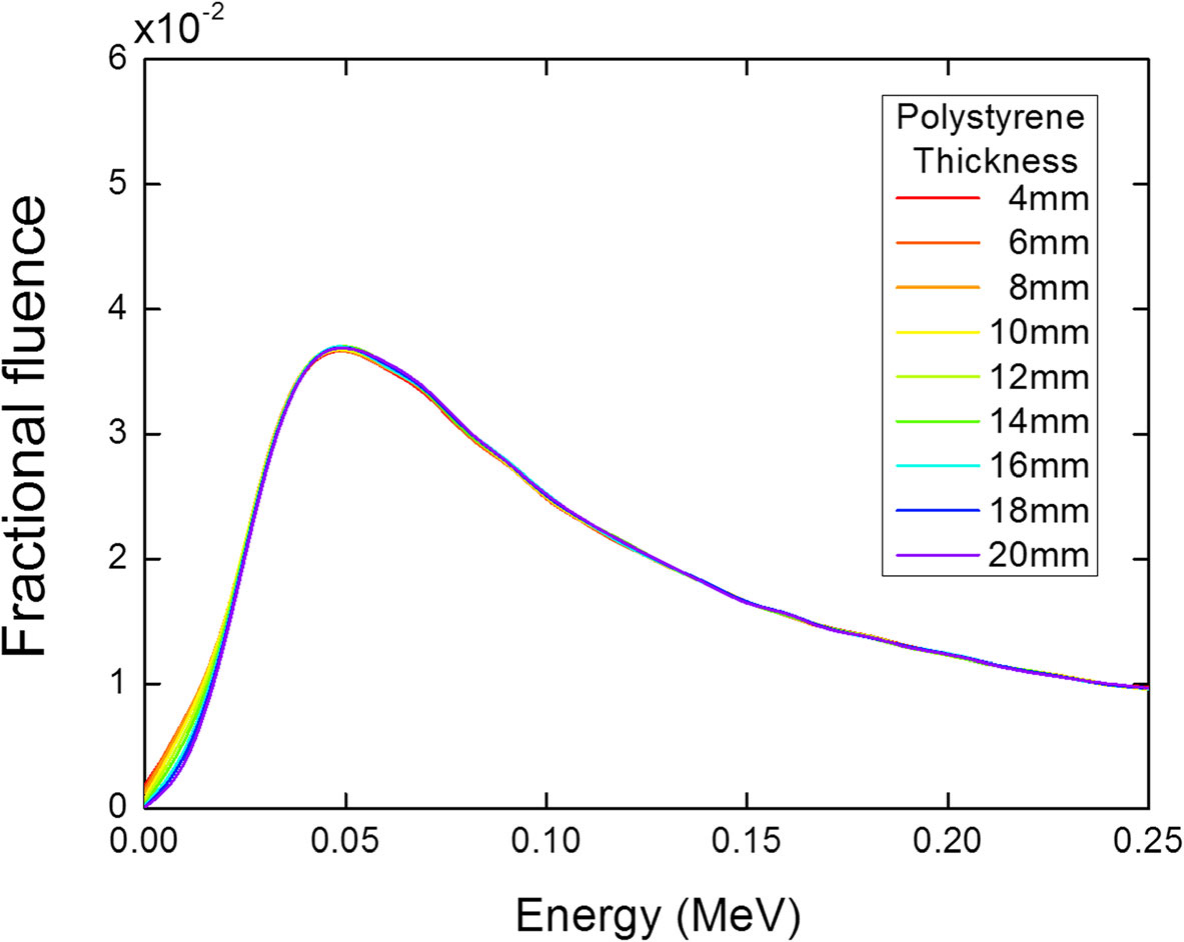
Relative low-energy photon spectra for the aluminum target beams with various polystyrene thicknesses

Table 3 lists the Monte Carlo simulation calculated proportions of the low-energy (25–150 keV) photons and electrons generated by the 8 mm aluminum target and 6 MeV electron beam with respect to the polystyrene thickness. The ratio of the remaining incident electron beam gradually decreased as the polystyrene thickness increased, and the electrons were nearly eliminated at the polystyrene thickness of 20 mm with minimal effect on the ratio of low-energy photons. This result is shown in Fig. 2, where the low-energy photon spectra for the aluminum target beams with various polystyrene thicknesses were almost identical, except in the very low-energy region. Furthermore, these results confirmed the high stopping power and low photon generation efficiency of the polystyrene plate. In previous studies, polystyrene electron filters of various thicknesses were used to remove the remaining incident electrons, because low-Z targets placed in the linac head carousel are not thick enough to stop all primary electrons [2, 7, 16, 22]. Low-Z target material types and thicknesses are difficult to determine owing to the continuous slowing down approximation (CSDA) range to a required tolerance within the confined space of the carousel. Even if a low-Z target is manufactured and installed in accordance with the carousel specification, a polystyrene filter is required. Conversely, the external aluminum target with a polystyrene plate used in this study requires simple machining and contains minimal installation constraints. Based on the results of the simulation, a 20 mm thick polystyrene plate was chosen as the electron filter, customized and placed under the 8 mm aluminum target.

**Table 3:** Monte Carlo simulation calculated percentages of photons in the energy range of 25–150 keV, and electrons generated by an aluminum target beam with various polystyrene thicknesses

Thickness (mm)	Proportion (%)	
	20–150 keV photons	Electrons
None	34.4	35.8
4	34.6	26.1
6	34.7	20.3
8	34.7	14.4
10	34.8	9.3
12	34.8	5.3
14	34.8	2.7
16	34.7	1.4
18	34.7	0.8
20	34.7	0.5

### B. Validation of the Al-target beam

**Fig. 3:**
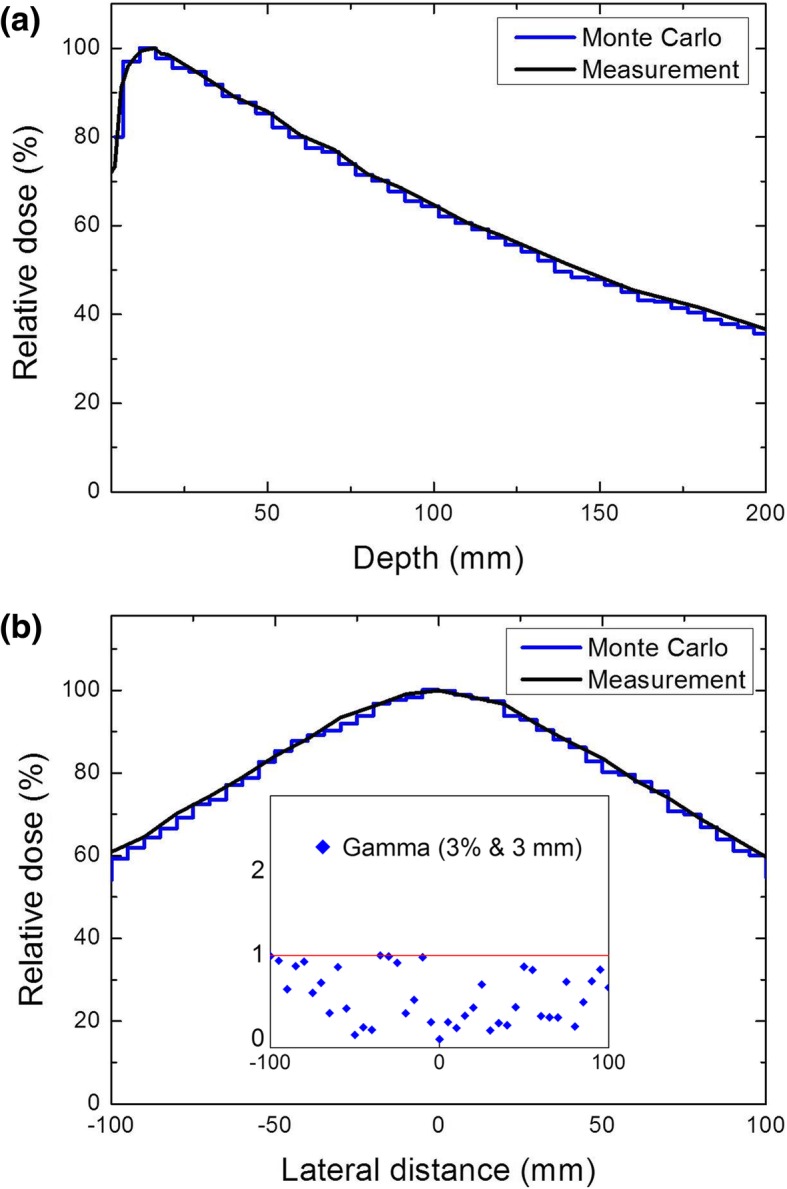
**a** Percentage depth dose curves obtained by measurement and modelling for the Al-target beams for a field size of 20 × 20 cm^2^ at a source-surface-distance of 100 cm, (**b**) lateral profiles of measured and modelled for the Al-target beams at a depth of 1.5 cm and field size of 20 × 20 cm^2^ at a source-surface-distance of 100 cm, the gamma evaluation represented in the inset image

To validate the Monte Carlo simulation, in terms of the Al-target beam quality, the modeled and measured PDDs and profiles in water were compared. Figure 3(a) shows the modeled and measured PDD curves of the Al-target beam for a field size of 20 × 20 cm^2^ and an SSD of 100 cm. The PDD curves were normalized to the maximum dose. The Monte Carlo simulation results of the PDD of the Al-target beam were consistent with the measured data to within 1.6%. Figure 3(b) shows lateral profiles for modeled and measured data for the Al-target beams at d_max_, for a field size of 20 × 20 cm^2^, at an SSD of 100 cm. The lateral profile curves were normalized to the dose values on the central axis. Figure 3(b) inset shows the corresponding gamma factor analysis for the modeled and measured profiles. For the criteria of 3% and 3 mm, all gamma values in the open beam region were less than one.

### C. Spatial resolution assessment

**Fig. 4:**
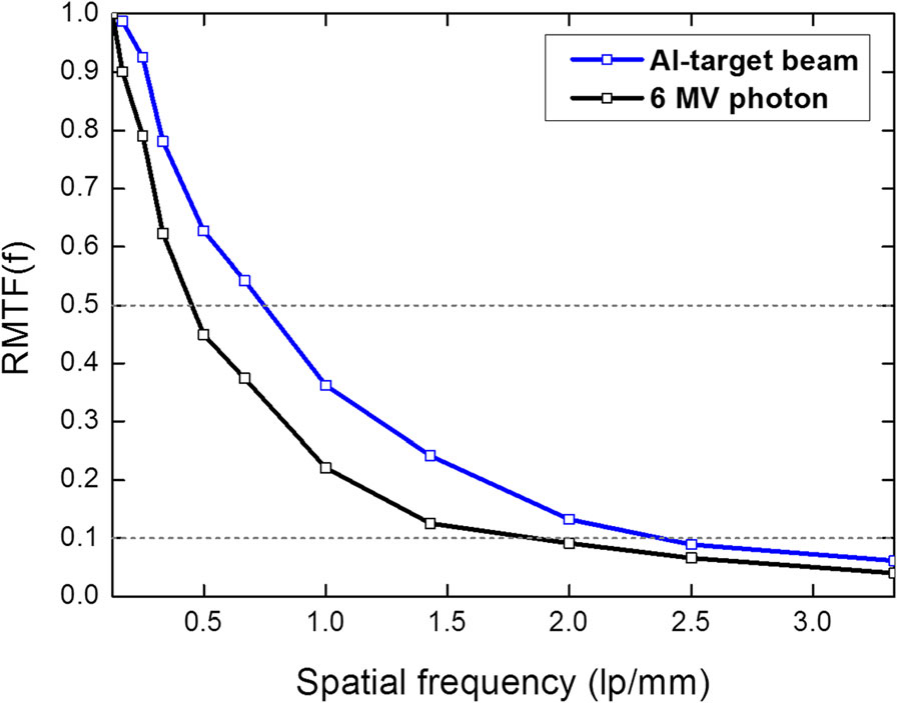
Relative modulation transfer function curves for 6 MV photon beam and the Al-target beam

**Fig. 5:**
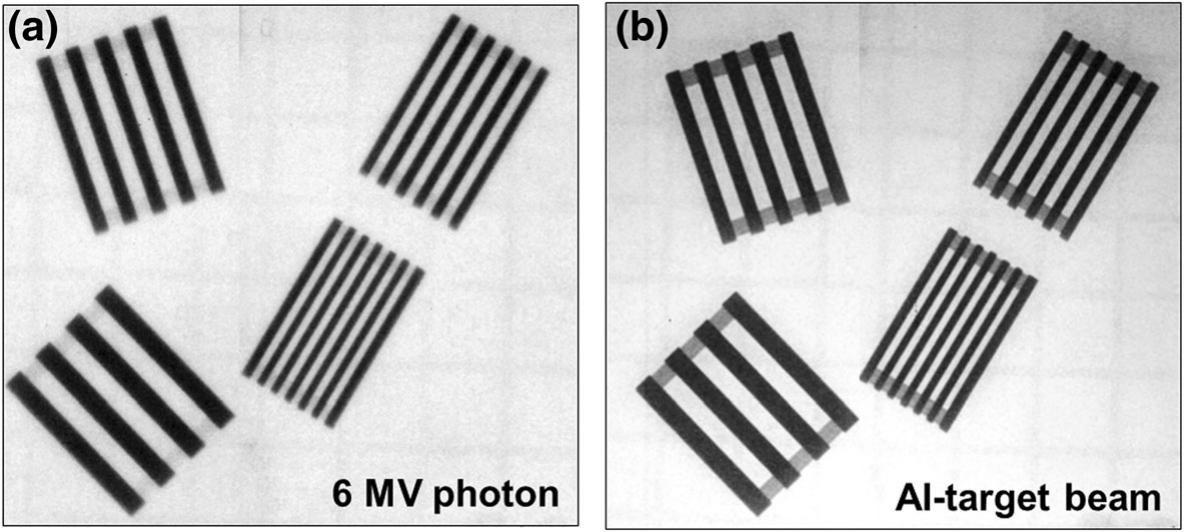
Diagonal line-pair images with spatial frequencies from 0.125 to 0.33 lp/mm for 6 MV photon beam and the Al-target beam

**Fig. 6:**
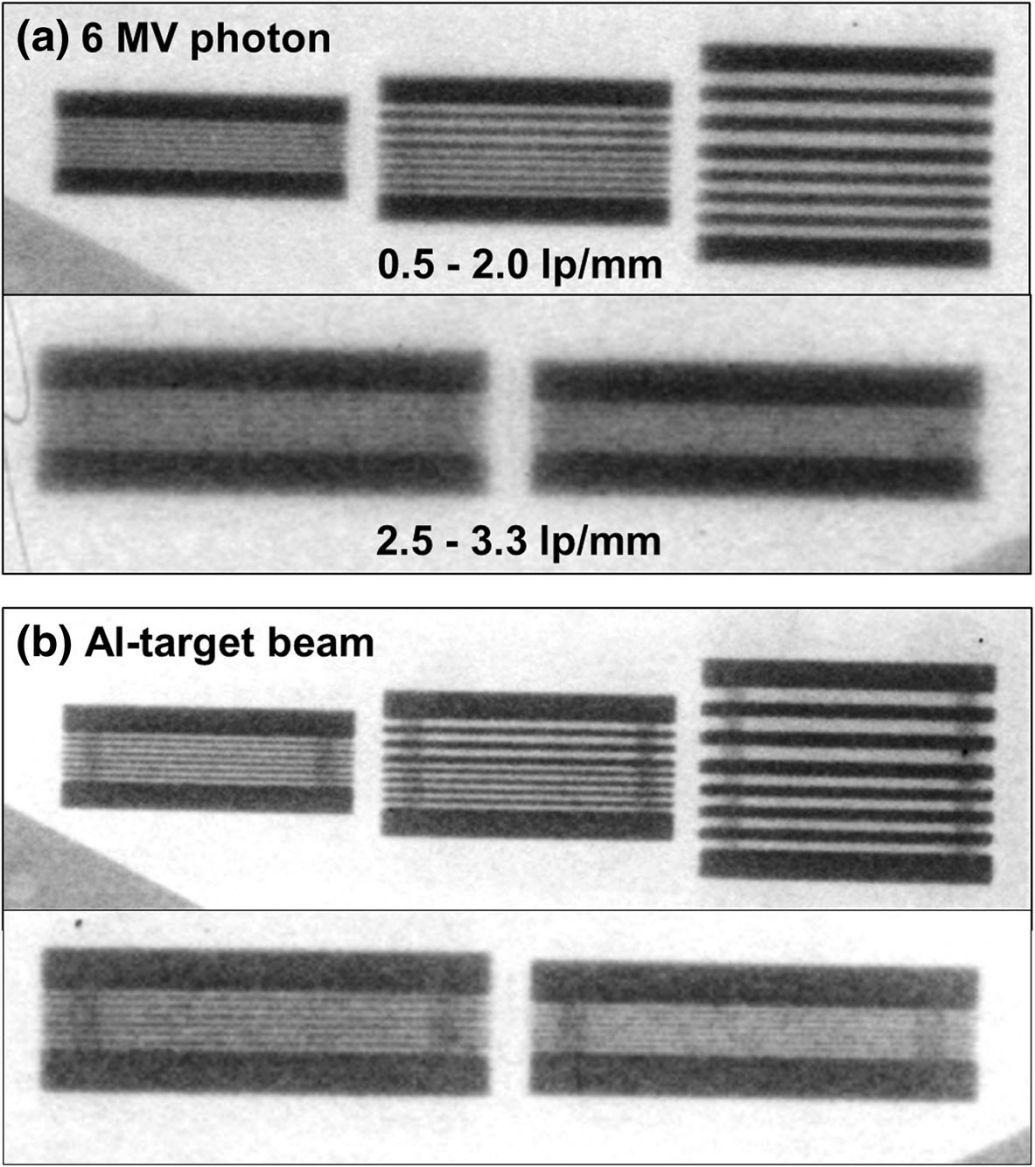
Horizontal line-pair images with spatial frequencies from 0.5 to 3.33 lp/mm for 6 MV photon beam and the Al-target beam

Figure 4 shows the MTF curves for the two imaging beams. The Al-target beam produced better MTF values at each spatial frequency than those of the 6 MV photon beam. The critical spatial frequency (*f*_50_) was also higher for the Al-target beam with a value of 0.745 lp/mm than those for the 6 MV photon beam with a value of 0.451 lp/mm. As shown in Fig. 5, all diagonal line pair images for the two imaging beams were clearly visible, and the lines and gaps were easily differentiated. As shown in Fig. 4, for the two imaging beams, the MTF values for the spatial frequency from 0.125 to 0.33 lp/mm were higher than the critical spatial resolution (*f*_50_) value. Figure 6 shows the horizontal line pair images of the two imaging beams for the spatial frequencies from 0.5 to 3.3 lp/mm. The Al-target beam produced darker and clearer line pair images than those of the 6 MV photon beam. For the 6 MV photon beam, the line pairs of the images were increasingly blurred as the spatial frequency increased, and distinction was not possible from a frequency of 2.0 lp/mm, where the MTF value fell below the limiting spatial resolution (*f*_10_) value. Conversely, the line pairs of the images for the Al-target beam remained distinguishable at 2.0 lp/mm, and the MTF value at this frequency was higher than the limiting spatial resolution value. These results suggested that the MTF of the detector system was dependent on the photon energy [16]. Thus, Al-target beams containing a large low-energy photon component are capable of producing higher MTF values than those of 6 MV photon beams. Figure 6 also shows the line pair images at the spatial frequencies in the range of 2.5 to 3.33 lp/mm for the two imaging beams, the Al-target beam produced the clear images; however, the lines and gaps were not distinguishable. This result was supported by the MTF curve shown in Fig. 4, where the MTF values for each spatial frequency in the range were lower than the limiting spatial resolution.

Previous studies using line-pair-type phantoms have reported that low-Z target beams could improve the spatial resolution compared to standard 6 MV photon beams [13, 16, 23]. Roberts et al. characterized spatial resolution using a full thickness 2 cm carbon target and a 4 MeV electron beam and found, using QC3 phantom line pair images, that an improvement in MTF was observed for the custom target beam. Connell and Robar investigated the effects of different beam generation parameters, such as the target atomic number, target thickness, and incident electron energy, on the spatial resolution and demonstrated that low-Z targets could produce high-resolution images as good as those acquired with 6 MV photon beams. However, in these previous studies, modifications of the linac head compositions and modulations of the incident electron energy were required, and thus, their findings are not easily applied to existing equipment. Conversely, in this study, the installation of external targets was simple because no modification of the linac head configuration was required, and thus, these results will be more applicable to the existing linacs if a detector suitable for the Al-target beams is designed.

### D. Contrast resolution assessment (contrast-detail distribution)

**Fig. 7:**
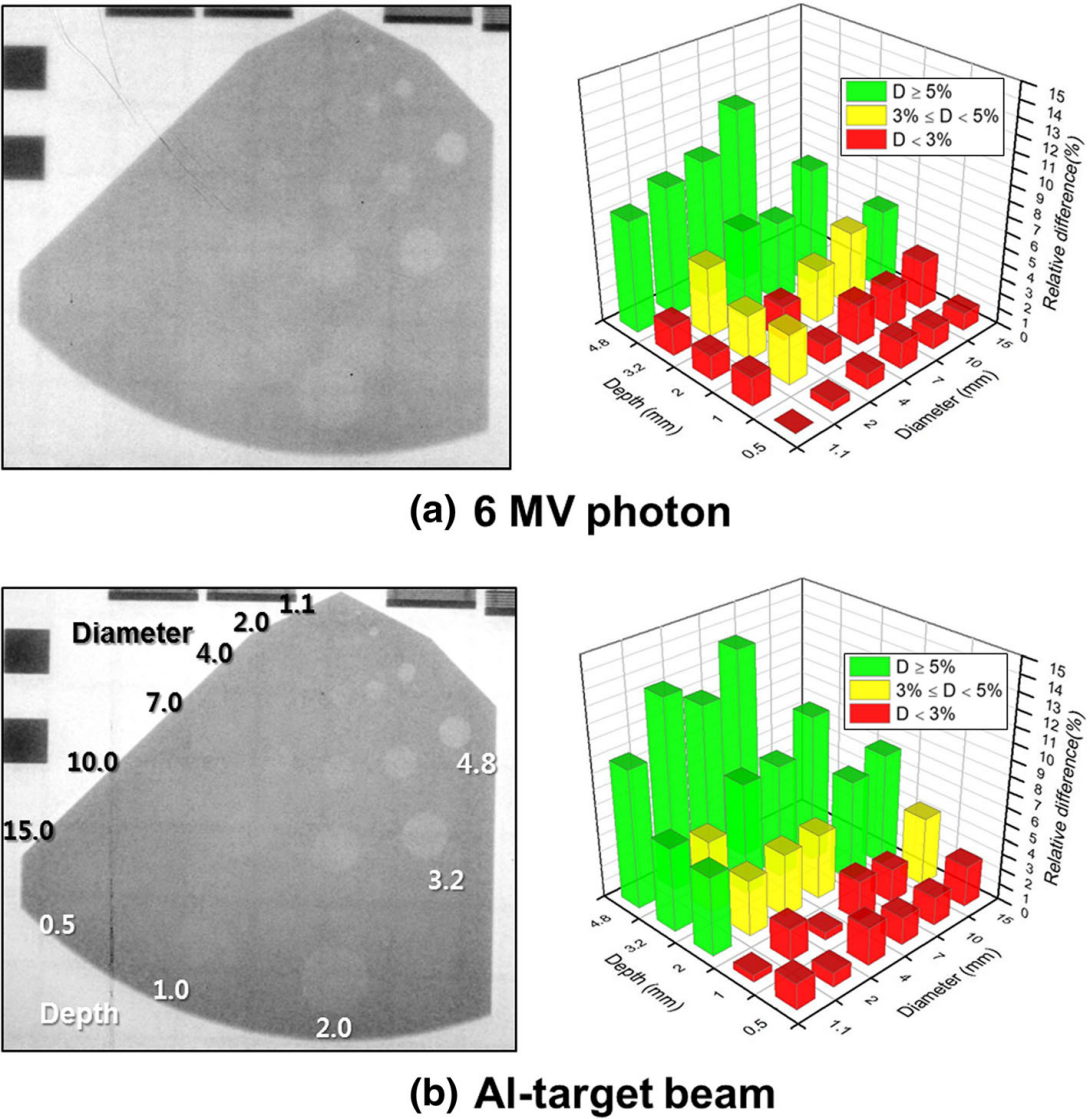
Contrast-detail element images and relative contrast difference distributions for 6 MV photon beam and the Al-target beam

Contrast resolution demonstrates the ability to detect subtle differences in grayscale in acquired images [21]. Figure 7 shows the contrast-detail test element images of the two imaging beams and also shows the relative contrast differences between the holes. Different hole depths and diameters indicate contrast and detail, respectively. The contrast-detail distributions are shown as color bars, where the green bars represent holes that can be distinguished with contrast differences ≥5%. The yellow bars represent barely distinguishable holes with contrast differences < 5% and ≥ 3%, and the red bars represent indistinguishable holes with contrast differences < 3%. The Al-target beam showed 11 green bars, 5 yellow bars, and 11 red bars. The 6 MV photon beam showed 8 green bars, 5 yellow bars, and 14 red bars. In the image of the Al-target beam, each hole was distinguishable with minimal noise or blurring, indicating that the Al-target beam resolved the smaller and lower-contrast objects better than that of 6 MV photon beams. This was attributed to the different attenuation coefficients of the Al-target beam and 6 MV photon beams, as shown by eq. (8). These findings show that the Al-target beams can improve the low-contrast resolution of the fine structures that cause small grayscale differences compared to the 6 MV photon beams.

### E. Future study

The next stage of this work involves the assessment of the image qualities produced using the Al-target beams with different object thicknesses. Because the low-energy component of the Al-target beams may be diminished when imaging thicker objects, a qualitative and quantitative analysis is required. A specific detector for the Al-target beams will be designed. However, because most MV portal imagers attached to the linac are optimized for MV photon beams, the Al-target beams cannot be used for imaging, and thus, detector design will be required.

## Conclusions

In this study, the Al-target beam was optimized by eliminating the remaining incident electrons using a 20 mm polystyrene filter, as determined by the Monte Carlo simulation. To validate the simulation, the PDDs and profiles were measured and compared with the modeled dose distributions. The phantom images acquired using the Al-target beams were analyzed quantitatively and qualitatively to evaluate the spatial and contrast resolutions. The Al-target beams produced sharper line pair images and a superior MTF curve than those of the 6 MV photon beam. In addition, the contrast-detail analysis demonstrated that the Al-target beams could resolve smaller and lower-contrast objects better than that of the 6 MV photon beam. In conclusion, the Al-target beams generated using an external aluminum target, which was installed on the upper accessory tray mount of a linac, provided better spatial and contrast resolutions than those of the 6 MV photon beam.

## Abbreviations

Al-target: Aluminum target
CSDA: Continuous slowing down approximation
IAEA: International Atomic Energy Agency
kV: kilovoltage
linac: linear accelerator
MTF: Modulation transfer function
MU: Monitor unit
MV: Megavoltage
OBI: On-board imagers
PDD: Percentage depth dose
SID: Source-to-imager distance
SSD: Source surface distance
